# Case Report of Overlapping Pubic Symphysis Dislocation Managed Nonoperatively

**DOI:** 10.7759/cureus.99034

**Published:** 2025-12-12

**Authors:** Majed S Alasbali, Mohammed S Alghamdi, Bader F Alsubie, Abdalaziz F Alamer, Abdullah M Alrafee

**Affiliations:** 1 Orthopedic Surgery, King Fahad Medical City, Riyadh, SAU; 2 Orthopedic Surgery, King Saud University, Riyadh, SAU

**Keywords:** locked pubic symphysis, nonoperative reduction, overlapping pubic symphysis, pelvis trauma, pubic symphysis dislocation, pubic symphysis reduction

## Abstract

Overlapping pubic symphysis dislocation (OPSD) is a rare form of pelvic injury typically caused by high-energy lateral compression forces, resulting in the entrapment of the pubic body against or behind the contralateral pubic ramus. The majority of reported cases have required open reduction and internal fixation due to the mechanical complexity of the injury. However, less invasive management strategies may be feasible in select cases.

We present a case of a 56-year-old male with diabetes who sustained an OPSD associated with a right sacral ala fracture, along with multiple nondisplaced rib and lumbar spine fractures, following blunt trauma. Urological evaluation, including CT urogram and ascending urethrogram, confirmed no bladder or urethral injury. Given the absence of posterior instability and urethral injury, this specific patient was managed conservatively under general anesthesia with fluoroscopic guidance. Manual reduction was successfully performed using a flexion, abduction, and external rotation maneuver of both hips, with anterior pressure on the symphysis.

The patient was instructed to perform non-weight-bearing mobilization for six weeks, followed by gradual mobilization. Follow-up imaging confirmed a maintained reduction without signs of pelvic instability or urological complications.

This case demonstrates that closed manual reduction can be a viable alternative to surgical intervention in selected cases of OPSD, particularly when there is no associated urethral injury or complex posterior pelvic ring disruption. Early recognition, appropriate imaging, skilled reduction under anesthesia, and maintained reduction at 18 weeks with no complications are critical in achieving favorable outcomes while minimizing surgical morbidity.

## Introduction

Locked symphysis pubis, or overlapping pubic symphysis dislocation (OPSD), is an infrequent type of pelvic injury due to lateral compression on the pelvic ring [1,2], causing the pubic body to displace and become entrapped against the contralateral side [2,3] or within the obturator foramen [1,3], resembling Tile classification B2.

It was first reported by Eggers in 1952 [4]. It is an uncommon injury and usually due to blunt trauma with hyperextension, adduction, and internal rotation, which result in pubic symphysis displacement anterior or posterior to the contralateral hemipelvis. In the literature, most cases were managed operatively with open reduction and internal fixation, but in our case, the injury was managed successfully with a manual reduction maneuver without surgical intervention.

 Although most reported OPSD cases require open reduction, we aim to demonstrate that closed reduction may suffice in select patients without posterior ring instability or urethral injury. We present our case to describe the less invasive method for the reduction of OPSD, where we rely only on the manual technique, sparing the patient's need for invasive surgical intervention and instrumentation, thus decreasing soft tissue injury and pain.

## Case presentation

**Figure 1:**
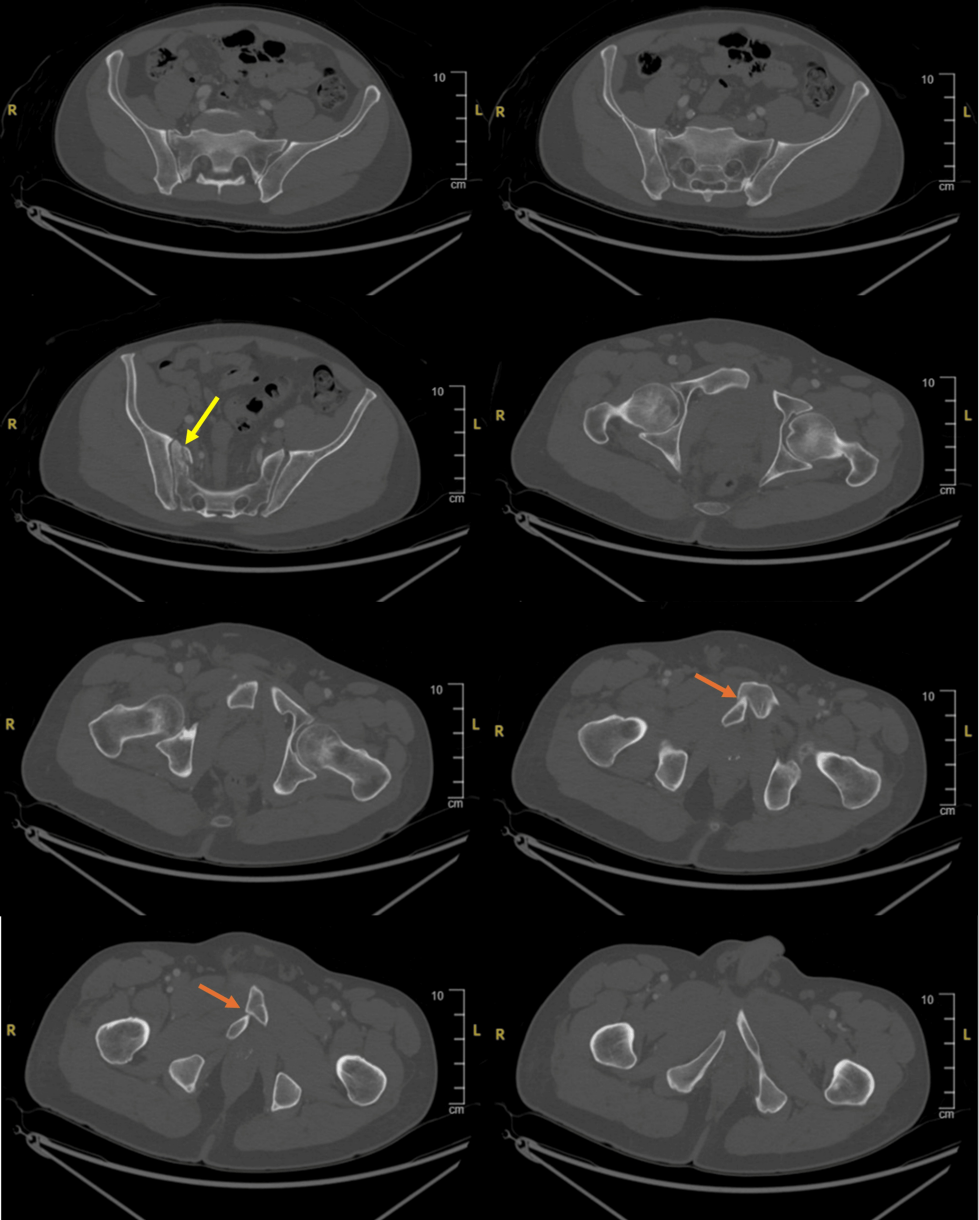
Pre-procedural axial CT scan showing overlapping pubic symphysis dislocation with entrapment of the pubic body and a comminuted right sacral ala fracture. The yellow arrow indicates the sacral injury. The orange arrow indicates the pubic injury. CT, computed tomography

**Figure 2:**
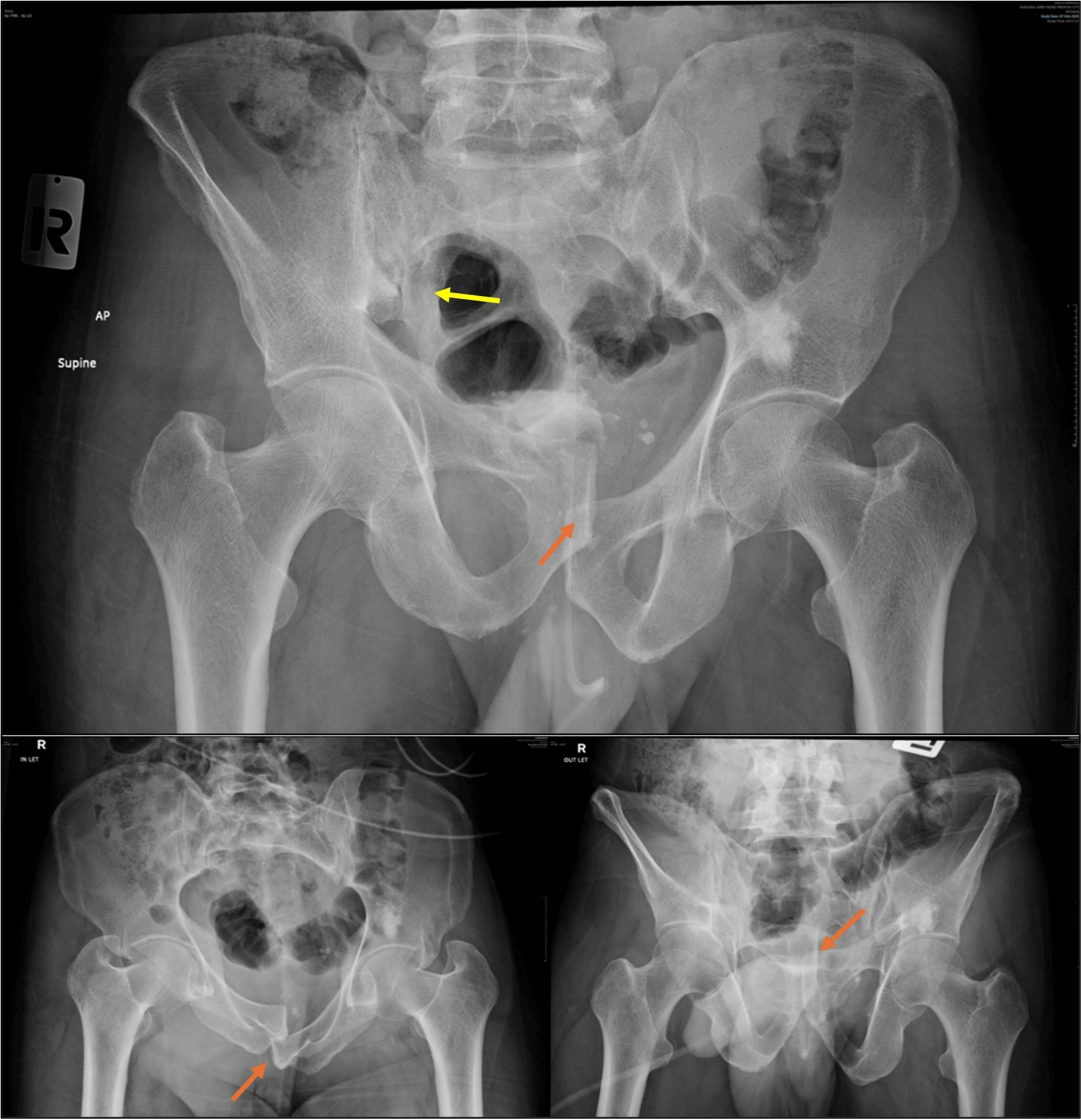
Pre-procedural AP, inlet, and outlet radiographs showing a right sacral ala fracture with overlapping pubic symphysis dislocation. The yellow arrow indicates the sacral injury. The orange arrow indicates the pubic injury. AP, anteroposterior

A 56-year-old male with diabetes mellitus presented to the Emergency Department of King Fahad Medical City with complaints of pubic pain, right buttock pain, and inability to bear weight after a heavy metal door fell on him. He sustained an overlapping pubic symphysis dislocation associated with a comminuted right sacral ala fracture (zone 1) according to the Denis classification system, with an intact neurological exam and preserved distal pulses. Upon urological assessment, there was no distended bladder and no blood at the meatus. A computed tomography urogram protocol showed no extravasation from the bladder, and a follow-up retrograde urethrogram demonstrated a well-distended bladder with no extravasation. The patient had multiple nondisplaced rib fractures on the right side involving the second, fourth, and fifth ribs, and on the left side involving the fourth, fifth, sixth, and seventh ribs. Additionally, there were lumbar spine fractures at L1 and L4, both of which were managed conservatively. Similarly, the pre-procedural assessment of the radiographic images showed a right sacral ala fracture with overlapping pubic symphysis dislocation (Figures 1-2).

**Figure 3:**
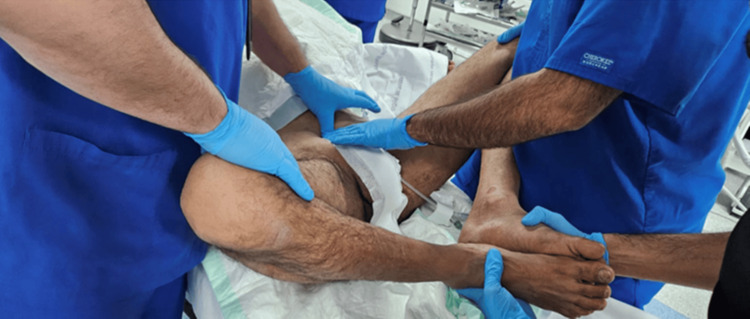
Sequential intraoperative photograph showing the closed reduction technique performed with both hips held in flexion and external rotation.

**Figure 4:**
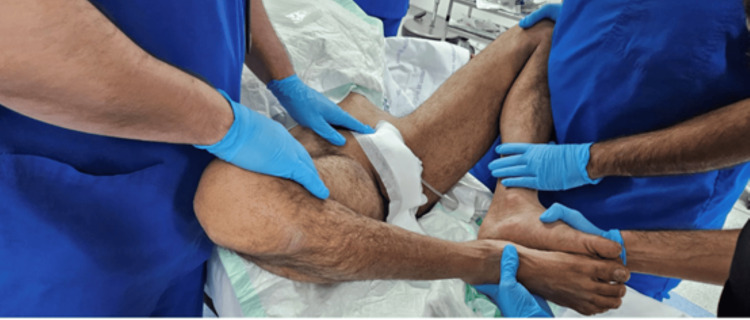
Sequential intraoperative photograph showing the closed reduction technique performed while maintaining hip abduction.

**Figure 5:**
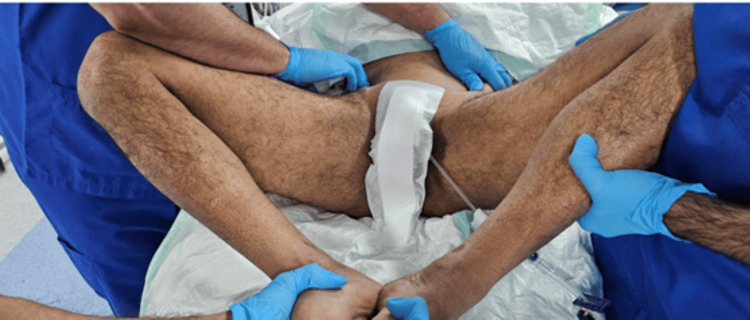
Sequential intraoperative photograph showing the closed reduction technique, performed with combined anterior and outward pressure over the iliac bone, resulting in successful reduction.

**Figure 6:**
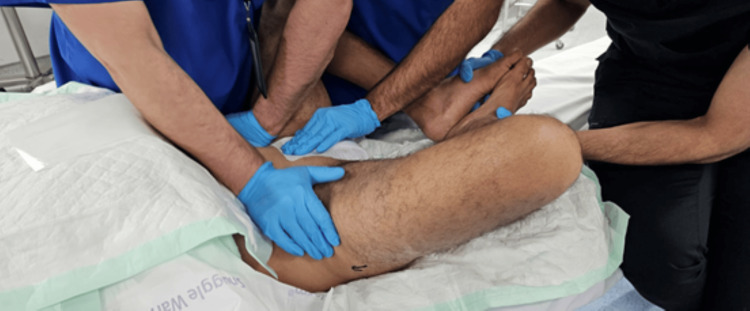
Sequential intraoperative photograph showing the closed reduction technique, with palpation of the pubic symphysis and application of pressure to assess the reduction.

**Figure 7:**
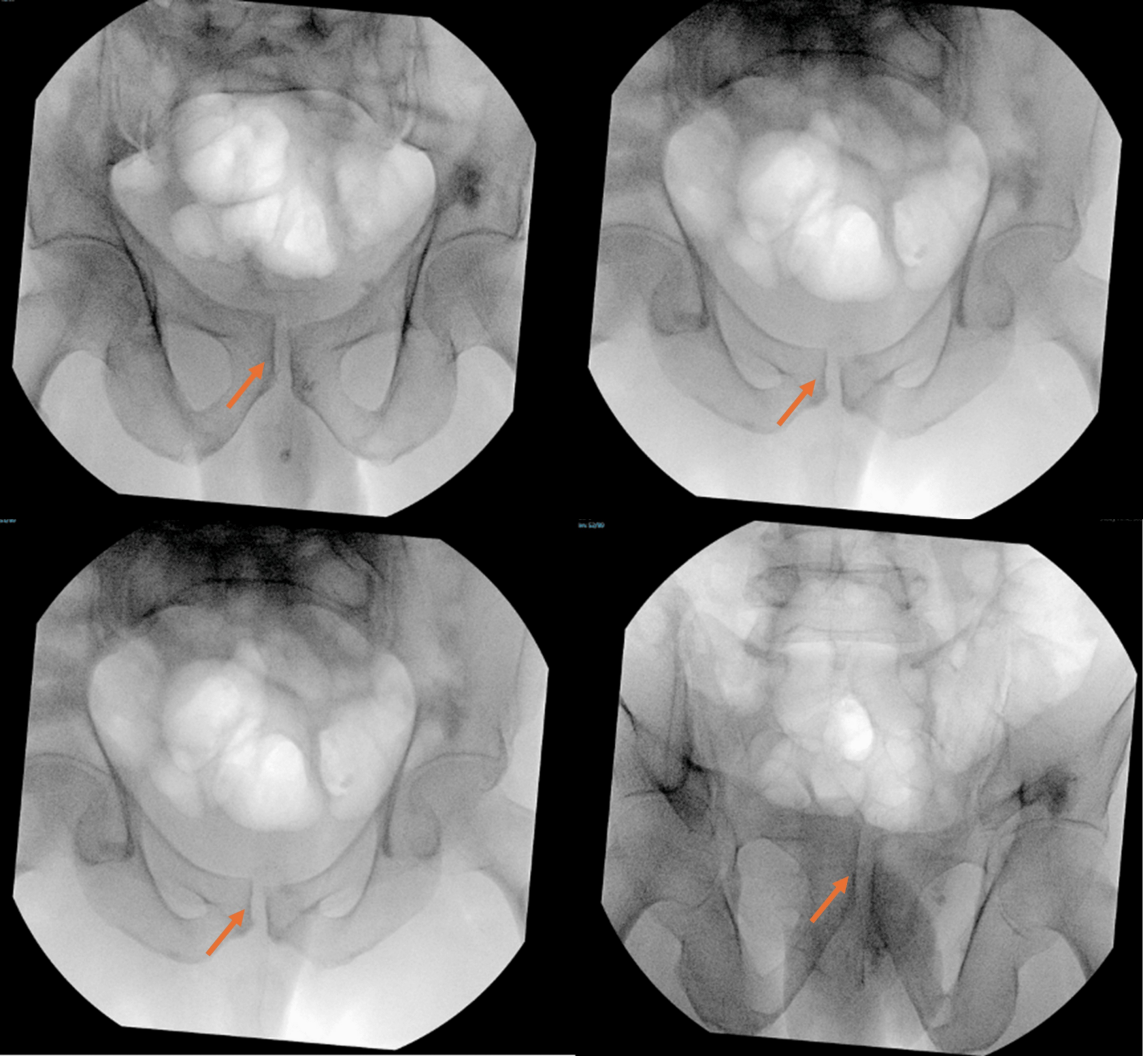
Intraoperative AP, inlet, and outlet fluoroscopic images showing realignment of the pubic symphysis. AP, anteroposterior

On day 2 post-trauma, the patient was taken to the operating room, positioned supine on a Jackson table, and, after induction of general anesthesia, a closed reduction maneuver was attempted by flexion, abduction, and external rotation of both hips while applying anterior and outward pressure with two hands over the bilateral anterior superior iliac spines, while the assistant palpated the pubic symphysis to assess the reduction, as shown in Figures 3-6. With C-arm fluoroscopy guidance, a closed reduction was achieved (Figure 7).

**Figure 8:**
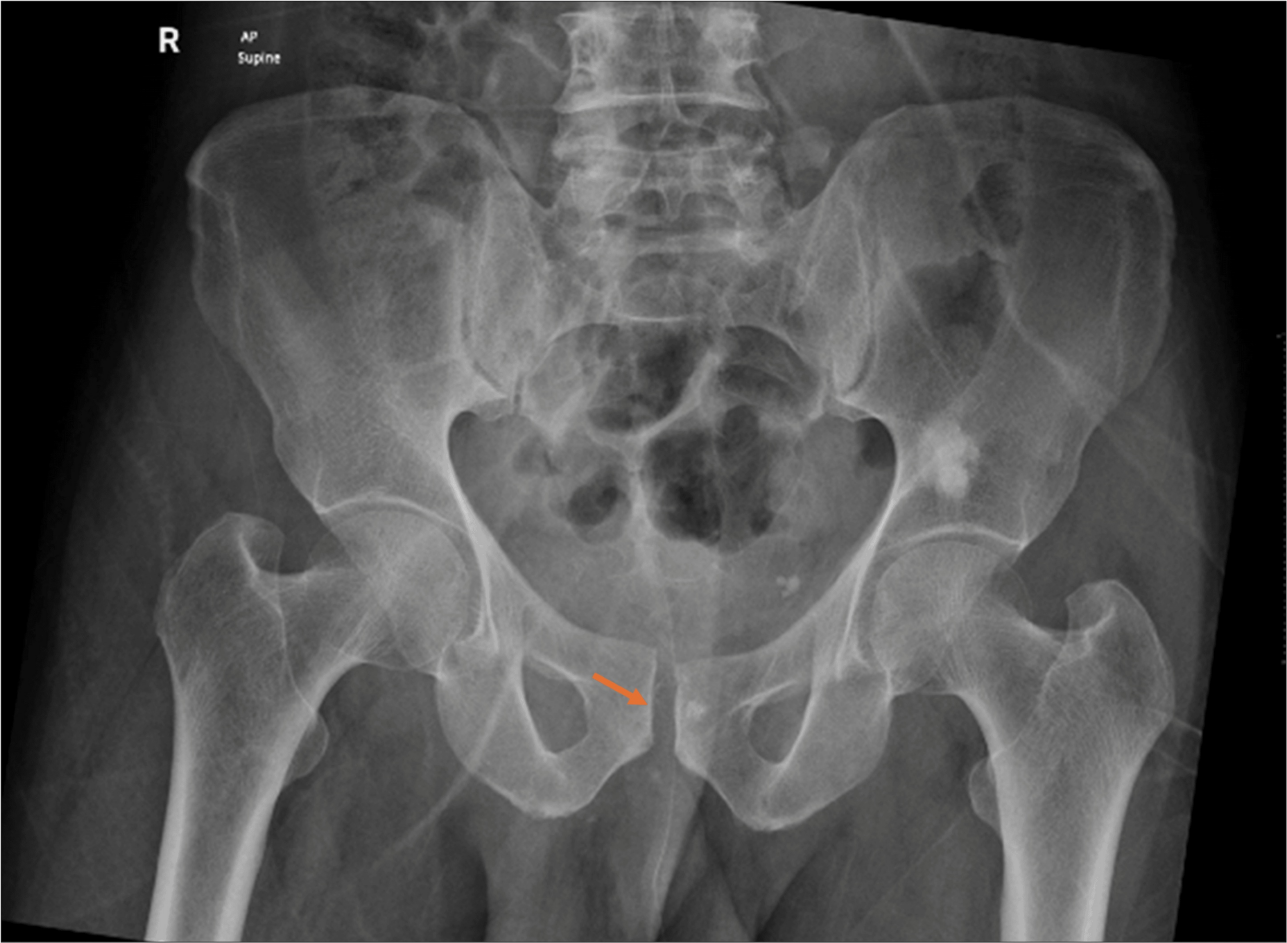
Post-reduction AP radiograph showing the reduced pubic symphysis. AP, anteroposterior

**Figure 9:**
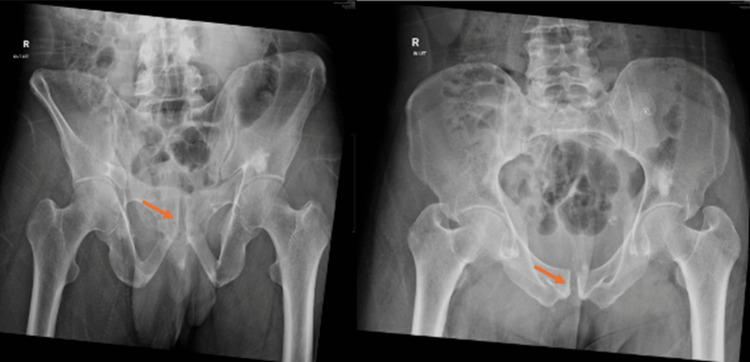
Post-reduction inlet and outlet radiographs confirming satisfactory reduction and alignment.

X-rays showed an acceptable reduction in anteroposterior (AP), inlet, and outlet views (Figures 8-9).

**Figure 10:**
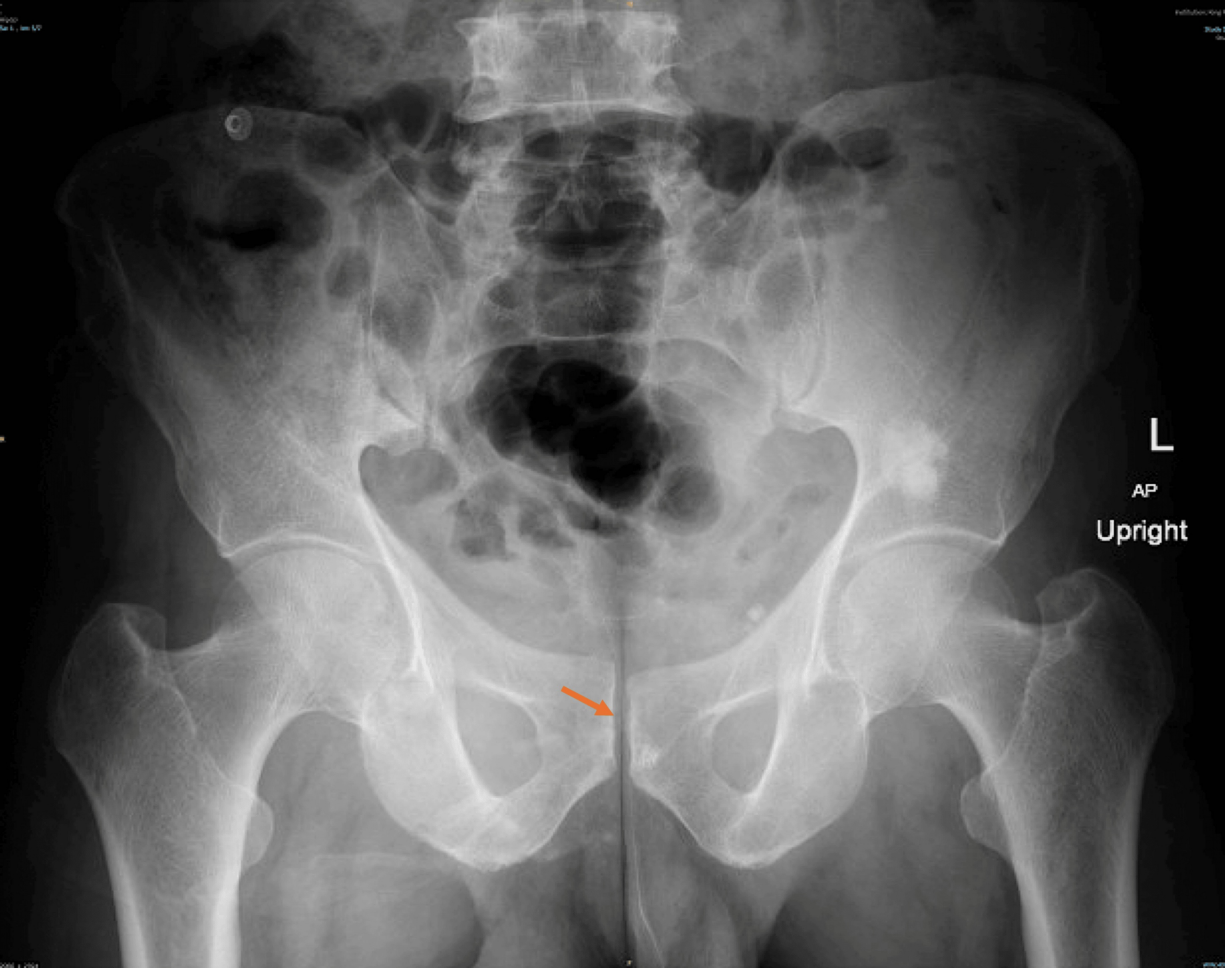
Weight-bearing X-ray confirms pelvic stability, reinforcing the clinical relevance of early mobilization. Radiograph shows a stable pelvis with maintained alignment on the second day after reduction. AP, anteroposterior

On the second day after reduction, weight-bearing radiographs showed a stable pelvis (Figure 10).

**Figure 11:**
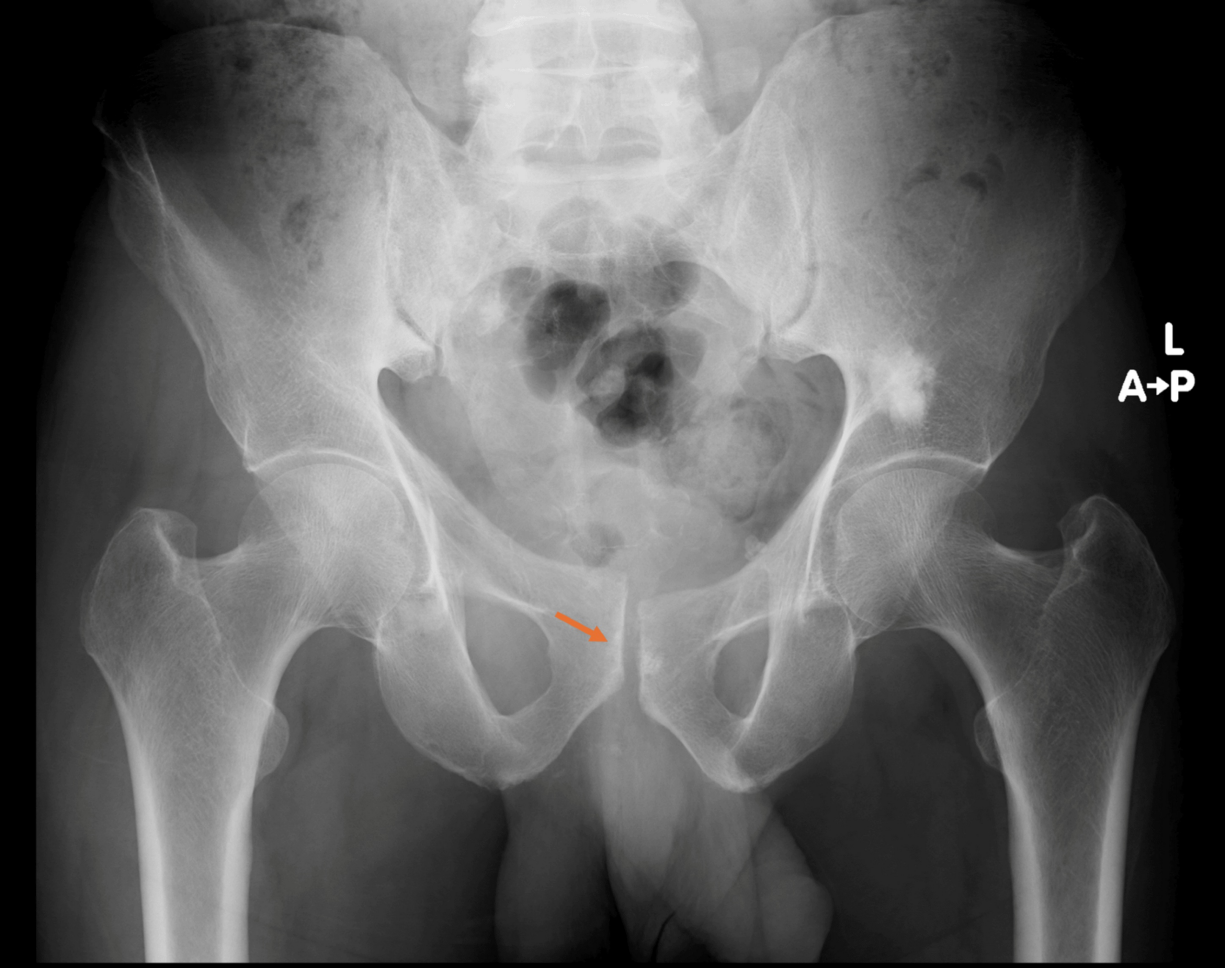
Serial follow-up pelvic radiographs after six weeks demonstrating maintained reduction, with an AP view showing stable alignment of the pubic symphysis. AP, anteroposterior

**Figure 12:**
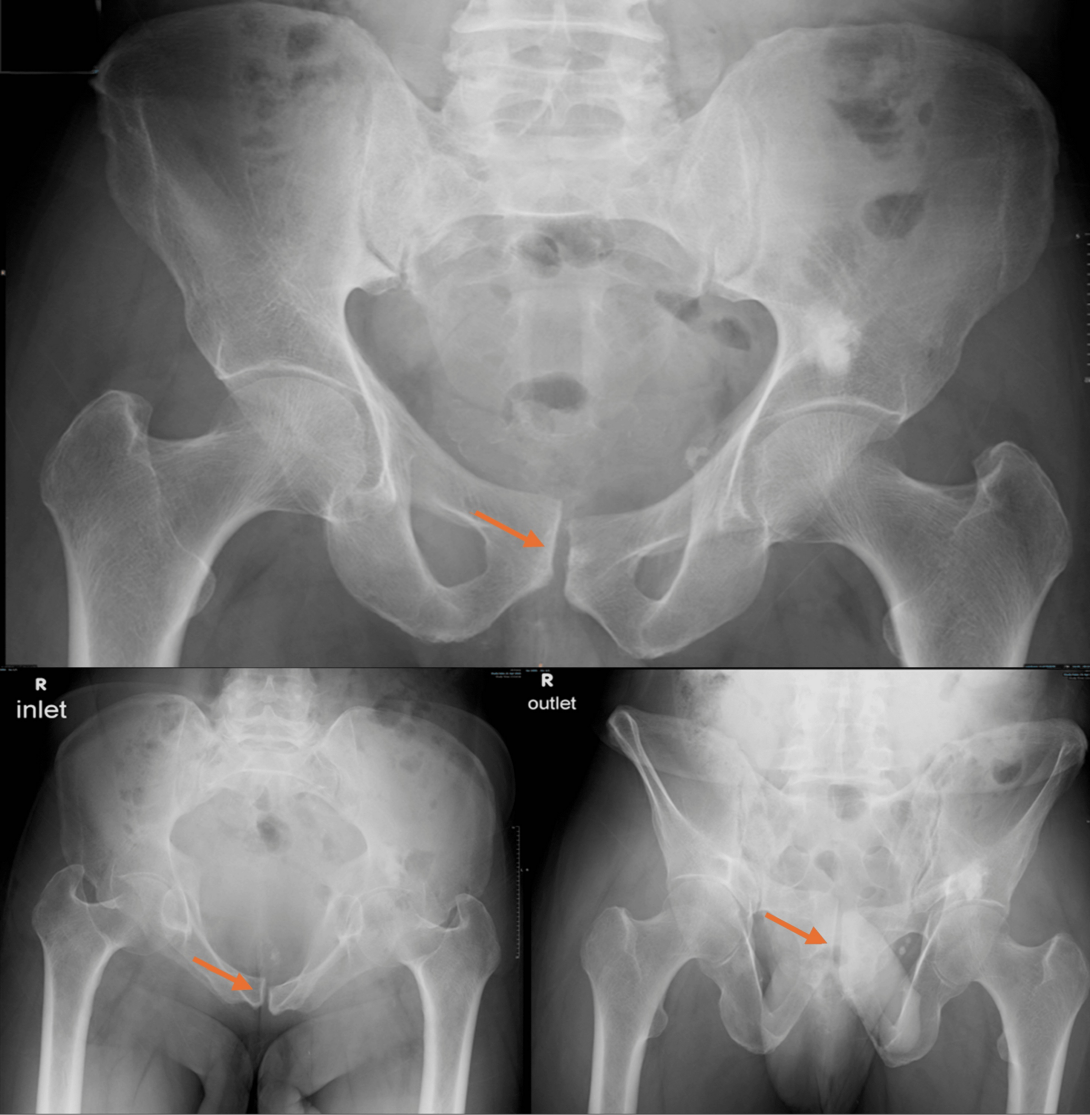
Serial follow-up pelvic radiographs after 10 weeks demonstrating maintained reduction in AP, inlet, and outlet views. AP, anteroposterior

**Figure 13:**
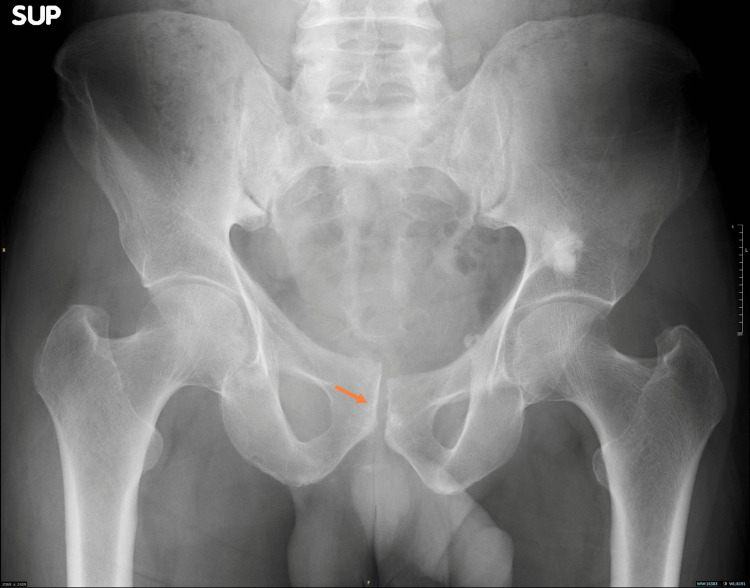
Serial follow-up pelvic radiographs after 18 weeks demonstrating maintained reduction in AP view. AP, anteroposterior

Postoperatively, the patient was managed with multimodal analgesia and physiotherapy focusing on functional transfer training, bed mobility, and strict non-weight-bearing for six weeks. At six weeks follow-up, he was allowed weight-bearing as tolerated. Follow-up evaluations at 6, 10, and 18 weeks with radiographs revealed maintained reduction (Figures 11-13). The patient reported no anterior or posterior pelvic pain and returned to his activities of daily living. He denied any symptoms of urinary dysfunction, confirming a favorable functional outcome.

## Discussion

OPSD is a rare and complex pelvic injury resulting from high-energy lateral compression forces. It typically involves the pubic body of one hemipelvis becoming entrapped against or behind the contralateral pubic ramus, or within the obturator foramen [2,4,5]. First described by Eggers in 1952, OPSD remains an uncommon entity, with only a limited number of cases reported in the literature [4].

Our case is notable for the successful closed manual reduction of an OPSD with an associated sacral ala fracture, managed without open surgery or internal fixation. Understanding the injury’s biomechanism, specifically how lateral compression entraps the pubic symphysis, enabled us to achieve reduction by reversing this mechanism. Our maneuver employed flexion, abduction, and external rotation to generate a lateral distraction force on the hemipelvis, combined with direct anterior pressure to disengage the entrapped symphysis. This conservative approach contrasts with the majority of published cases, in which surgical intervention was necessary due to the mechanical complexity of the injury or associated complications.

For instance, Chee and Ahmad reported a locked symphysis associated with iliac wing and femoral shaft fractures that required open reduction and internal fixation via an anterior pubic symphysis approach [2]. Likewise, Tadros et al. reported three cases of OPSD, each involving varying posterior pelvic injuries, all of which required open reduction and internal fixation [5].

In some reported severe cases, osteotomy of the contralateral superior pubic ramus was required for reduction when closed and standard open techniques failed. Rajput et al. and Basu et al. presented cases in which osteotomy was required to release the irreducible locked pubic ramus. However, long-term complications such as urethral stricture, sexual dysfunction, heterotopic ossification, and implant failure were noted, raising concerns about the morbidity associated with such invasive procedures [6, 3].

Pushpasekaran et al. used a distraction osteotomy in an 18-year-old patient and achieved mechanical realignment, although the patient developed a urethral stricture requiring later repair [7]. Similarly, Tsehaye and Teklu described an open approach in the setting of a concurrent acetabular fracture, again emphasizing the need for open reduction internal fixation in more complex injury patterns [8].

There is limited but important support in the literature for non-surgical management. Afshar and Koushkzari reported a case in which closed reduction under general anesthesia was sufficient, although the patient later had persistent urological complications due to urethral transection, so even nonoperative management is not without risk of further injury [1]. Notably, our patient had no signs of urethral or bladder injury on CT urogram or ascending urethrogram, and post-reduction follow-up demonstrated stable alignment with no complications or signs of pelvic instability.

Fergany et al. also emphasized the role of soft tissue entrapment as a cause of failed reduction [9]. However, their case required surgical bladder dissection, highlighting the importance of urological evaluation before attempting reduction, which was adequately addressed in our workup. A favorable outcome was noted in this case after reduction, as pain decreased, gait normalized, and the patient was able to return to work with normal urological function. 

## Conclusions

Our patient benefited from a well-executed closed reduction maneuver under fluoroscopic guidance. The technique involved flexion, abduction, and external rotation of the hips while applying anterior and outward pressure over the bilateral anterior superior iliac spines. This avoided the risk of surgical exposure and its potential risks like infection, iatrogenic injury, and prolonged hospitalization. Post-reduction management with non-weight-bearing followed by gradual mobilization resulted in favorable early outcomes.

This case shows that closed reduction can be a viable and safe alternative in selected cases of OPSD, particularly when there is no entrapment in the obturator canal, no urethral injury, and no complex posterior ring instability, confirming radiologic and functional stability up to 18 weeks. The key elements for success include adequate imaging to rule out soft tissue incarceration, prompt reduction under anesthesia, and proper follow-up. Hence, this case underscores that fluoroscopy-guided closed reduction may provide a safe and effective management option in OPSD when careful selection and imaging evaluation are performed. 

## References

[1] Afshar A, Koushkzari M (2015). Overlapped pubic symphysis: a case report and review of the literature. Arch Bone Jt Surg.

[2] Chee WH, Ahmad AR (2018). Locked pubic symphysis: a case report and review of literature. JUMMEC.

[3] Basu A, Shukla N, Velagada S, Behera S (2023). Mid-term follow-up of superior pubic ramus osteotomy in locked symphysis pubis with urethral injury: a case report. Chin J Traumatol.

[4] Eggers GWN (1952). Dislocations of the os coxae. Am J Surg.

[5] Tadros AM, Lunsjo K, O'Brien P (2009). Overlapping dislocation of the pubic symphysis: report of three cases and review of the literature. Arch Orthop Trauma Surg.

[6] Rajput R, Pal KK, Goel AK, Mandal A (2021). Irreducible overlapping pubic symphysis dislocation managed with distraction osteotomy of the contralateral superior pubic ramus: a rare case report. J Orthop Case Rep.

[7] Pushpasekaran N, Thampy S, Khaleel VM, Joseph S (2020). Treatment of locked pubic symphysis by distraction osteotomy of the superior pubic ramus: a case report. JBJS Case Connect.

[8] Tsehaye M, Teklu D (2021). Case report on locked pubic symphysis with concomitant ipsilateral acetabular fracture. Int Med Case Rep J.

[9] Fergany A, Khalifa AA, Mokhtar FA, Farouk O (2025). Irreducible locked symphysis pubis disruption caused by incarcerated urinary bladder in a 14-year-old boy: a case report and review of the literature. Orthop Res Rev.

